# Role of N–epsilon- carboxy methyl lysine, advanced glycation end products and reactive oxygen species for the development of nonproliferative and proliferative retinopathy in type 2 diabetes mellitus

**Published:** 2013-01-28

**Authors:** Subhadip Choudhuri, Deep Dutta, Aditi Sen, Imran Hussain Chowdhury, Bhaskar Mitra, Lakshmi Kanta Mondal, Avijit Saha, Gautam Bhadhuri, Basudev Bhattacharya

**Affiliations:** 1 Department of Biochemistry, Dr. B C Roy Post Graduate Institute of Basic Medical Education and Research (IPGME&R), Kolkata, India; 2 Regional Institute of Ophthalmology, Kolkata, India; 3 Department of Endocrinology & Metabolism, IPGMER & SSKM Hospital, Kolkata, India; 4 Department of Pathology, Midnapore Medical College, Paschim Midnapore, India

## Abstract

**Purpose:**

The aim of the present study was to evaluate the collective role of N-epsilon–carboxy methyl lysine (*N*^ε^-CML), advanced glycation end-products (AGEs), and reactive oxygen species (ROS) for the development of retinopathy among type 2 diabetic subjects.

**Methods:**

Seventy type 2 diabetic subjects with nonproliferative diabetic retinopathy (NPDR), 105 subjects with proliferative diabetic retinopathy (PDR), and 102 patients with diabetes but without retinopathy (DNR) were enrolled in this study. In addition, 95 normal individuals without diabetes were enrolled as healthy controls in this study. Serum and vitreous *N*^ε^-CML and AGEs were measured by enzyme-linked immunosorbent assay. The peripheral blood mononuclear cell (PBMC) ROS level was measured by flow cytometric analysis. Serum and PBMC total thiols were measured by spectrophotometry.

**Results:**

Serum AGEs and *N*^ε^-CML levels were significantly elevated in subjects with PDR (p<0.0001) and NPDR (p=0.0297 and p<0.0001, respectively) compared to DNR subjects. Further vitreous AGEs and *N*^ε^-CML levels were found to be significantly high among PDR subjects compared to the control group (p<0.0001). PBMC ROS production was found to be strikingly high among NPDR (p<0.0001) and PDR (p<0.0001) subjects as compared to the DNR group. Serum and PBMC total thiol levels were remarkably decreased in NPDR (p<0.0001 and p=0.0043, respectively) and PDR (p=0.0108 and p=0.0332 respectively) subjects than those were considered as DNR.

**Conclusions:**

Our findings suggest that *N*^ε^-CML and ROS are the key modulators for the development of nonproliferative retinopathy among poorly controlled type 2 diabetic subjects. Furthermore, AGEs under persistent oxidative stress and the deprived antioxidant state might instigate the pathogenic process of retinopathy from the nonproliferative to the proliferative state.

## Introduction

Diabetic retinopathy (DR), a form of microangiopathy, is one of the leading causes of blindness around the world [1]. Chronic uncontrolled hyperglycemia has been suggested as a major influencing factor for retinal microvascular pericyte and endothelial cell (EC) dysfunction in type 2 diabetes mellitus (DM) [2,3]. Among the different biochemical pathways implicated in the pathogenesis of DR, the process of formation and accumulation of advanced glycation end-products (AGEs) and their modes of action have been considered as major initiators of retinal microvascular complications in type 2 DM [4,5]. AGEs are nonenzymatically glycated and oxidized proteins or lipids that accumulate in the vessel wall, where they may perturb vascular endothelial and pericyte cell structure and function [6]. In vitro studies have shown that N-epsilon–carboxy methyl lysine (*N*^ε^-CML) and other AGEs are toxic to retinal pericytes and have a deleterious influence on pericyte cell survival [7-9]. In particular, *N*^ε^-CML, the most prevalent AGE, interacts with receptors of AGE (RAGE), which in turn activates signal transduction pathways that leads to the expression of proinflammatory genes [10,11]. AGEs may also modify the action of free radicals, and may thus impact on the function of intracellular proteins via the interaction with RAGE [12]. AGE-bound RAGE worsens microvascular dysfunction through increased microvascular EC permeability and increased production of reactive oxygen species (ROS) through the activation of reduced nicotinamide adenine dinucleotide phosphate (NADPH) oxidase [13,14].

Increased production and the consequent ineffective elimination of ROS by a poor cellular antioxidant system is one of the major causes of the development of oxidative stress among patients with type 2 DM [15]. A family of multisubunit (intracellular and membrane-bound) NADPH oxidases appears to be the predominant contributor for endothelial and pericyte ROS production, which have been increasingly appreciated to have a detrimental role in retinal microvascular pathophysiology [16-18]. In this regard, total thiols (–SH) that exist extra- as well as intracellularly in free (reduced glutathione) or bound form (protein-bound thiol), reduce the highly reactive super oxide radicals and thereby maintain intracellular homeostasis [19,20]. However, the involvement of endothelial and pericyte cell dysfunction in the pathogenesis of DR still remains enigmatic. Comparatively fewer amounts of data and information are available on AGE activation along with the ROS generation rate and antioxidant status at different stages of DR.

The aim of the present study was to investigate whether AGE and their late oxidative product *N*^ε^-CML levels in normal individuals without diabetes (healthy control; HC), type 2 DM patients without retinopathy (DNR), and patients with DR, i.e., both nonproliferative DR (NPDR) and proliferative DR (PDR), were significantly different. We focused on serum and vitreous levels of *N*^ε^-CML to evaluate whether these glycoxidized ligands of RAGE intimately associated with intracellular ROS formation and in turn initiate the pathogenic process of retinopathy. We also evaluated the relationship between AGEs and oxidative stress and their combined impact on different stages of DR, i.e., in NPDR and PDR, by measuring the following parameters:

I) Serum levels of total AGEs and *N*^ε^-CML were measured among different study subjects. Further, total AGE and *N*^ε^-CML were analyzed in vitreous from PDR and controls. II) The peripheral blood mononuclear cell (PBMC) ROS levels were analyzed to evaluate oxidative stress in different study subjects. Antioxidant status at different stages of DR was determined by the measurement of serum and PBMC total thiol level.

## Methods

### Study subjects

One-hundred and five PDR subjects (mean age=53.4±8.15 years), 70 NPDR subjects (mean age=52±8.8 years), 102 DNR subjects (mean age=53.1±7.86 years), and 95 HC subjects (mean age=51.5±7.24 years) were enrolled in this cross-sectional study. A presence of coronary artery disease (CAD) or a strong family history of CAD, hypertension (defined according to the new criteria i.e., systolic blood pressure >140 mmHg and diastolic blood pressure >90 mmHg), peripheral vascular disease, recent acute infection, thrombotic events, urinary microalbumin >300 mg/day, prediabetes (fasting blood glucose >100 mg/dl but <126 mg/dl and postprandial blood glucose >140 mg/dl but <200 mg/dl), and ocular disorder (glaucoma, Eale’s disease, branch retinal venous occlusion, etc.) were considered exclusion criteria for this study.

The samples were obtained from the retina clinic of Regional Institute of Ophthalmology and diabetic clinic of Institute of Postgraduate Medical Education and Research, Kolkata. All the subjects enrolled in this study belonged to same geographical area (Gangetic Delta, eastern India). Written informed consent was collected from each patient according to the Declaration of Helsinki and was approved by ethical committee of the institute.

Age, sex, and blood pressure were matched within the study groups. DM was diagnosed according to the World Health Organization criteria [21]. We investigated the glycemic status of all diabetic subjects by the oral glucose tolerance test and glycosylated hemoglobin (HbA1c %) test. None of the study subjects were on insulin treatment during the study period. PDR and NPDR were diagnosed by dilated fundus examination with slit-lamp biomicroscopy by ±90D and three-mirror lens seven field digital fundus photography with fluorescence angiography. Grading of the retinopathy was carried out according to a modified early treatment DR study.

### Sample collection and processing

Study subjects were advised to be in a 12 h strict fasting state before collection of blood samples. Thereafter, 15 ml venous blood samples were drawn. A 10 ml blood sample was collected in a heparinized tube for PBMC isolation and a 5 ml sample was taken in a clot vial to obtain serum. Finally, serum samples were collected in CryoCube vials for total AGE, *N*^ε^-CML, and thiol assay.

Mononuclear cells from peripheral whole blood were obtained from 10 ml heparinized blood by using Histopaque 1077 separating media (Sigma Aldrich, St Louis, MO) density gradient for 40 min at 150 ×g and 20 °C, as previously described [22]. PBMCs were further subjected to centrifugation at 200 ×g for 10 min and washed twice with 1× PBS (pH 7.2). Then, 5×10^5^ and 5×10^6^ cells were pelleted into two different tubes and resuspended in 1× PBS (pH 7.2) for the estimation of ROS and intracellular thiol, respectively.

Vitreous samples were drawn by three-port pars plana vitrectomy from 45 PDR and 36 control subjects (normal vitreous was collected from study subjects undergoing emergency vitrectomy after an accident). Only AGEs and *N*^ε^-CML levels were measured from the vitreous fluid of PDR subjects and from those considered nondiabetic controls. Two hundred microliters of undiluted vitreous gel was excised from the midvitreous using a vitreous cutter and carefully aspirated into a handheld sterile syringe attached to the suction port of the vitrectomy probe. Immediately after collection, the vitreous samples were kept on ice and centrifuged at 8,950 ×g for 15 min at 4 °C. After centrifugation, the supernatant was aspirated and stored at −20 °C for immediate use.

### Measurement of total advanced glycation end-products from serum and vitreous

The AGE protein adducts present in the sample were measured by enzyme-linked immunosorbent assay by using the Cell Biolabs kit (catalog No. STA 317; Cell Biolabs, San Diego, CA). AGEs present in the sample were probed with an anti-AGE polyclonal antibody, followed by a horseradish peroxidase–conjugated secondary antibody. The AGE protein adduct content in the sample was determined by comparison with a standard curve prepared from AGE–bovine serum albumin (BSA) standards ranging from 0.25 to 5 µg/ml. The absorbance of the final color product was read at 450 nm as the primary wavelength using a Bio Rad multiplate reader (Model 680, Bio Rad, Laboratories, Hercules, CA) against the reduced BSA standard as the absorbance blank. The AGE–BSA provided in the kit was prepared by reacting BSA with glycolaldehyde, followed by extensive dialysis and column purification. AGE – BSA contains CML, pentosidine, and other AGE structures.

### Measurement of N-epsilon carboxy methyl lysine from serum and vitreous

CML protein adducts present in the sample were measured by enzyme-linked immunosorbent assay by using the Cell Biolabs kit (catalog No. STA 316). CML present in the sample was probed with an anti-CML antibody, followed by a horseradish peroxidase–conjugated secondary antibody. The CML protein adduct content in the sample was determined by comparison with a standard curve prepared from CML–BSA standards ranging from 0.035 to 2.2 ng/ml. The absorbance of the final color product was read at 450 nm as the primary wavelength by a Bio Rad multiplate reader (Model 680) against the reduced BSA standard as the absorbance blank.

### Measurement of peripheral blood mononuclear cell reactive oxygen species

Intracellular ROS generation in mononuclear cells was measured by ROS-sensitive cell-permeable dye 2´7´ dihydrodichlorofluorescein diacetate (2´7´ H_2_DCF-DA), which in the presence of ROS was oxidized to highly fluorescent 2´7´- dichlorofluorescein (2´7´ DCF) in the cell. Production of intracellular ROS is directly proportional to the oxidation of 2´7´ H_2_DCF-DA, and thereby elevates the cellular fluorescence level. Pelleted cells (5x10^5^) were washed twice with 1× PBS (pH 7.2) by centrifuging at 1,430 ×g for 5 min and cells were resuspended in 500 µl of 1× PBS (pH 7.2). Thereafter, cells were incubated with 20 µm 2´7´ H_2_DCF-DA for 30 min at 37 °C. Finally, the cells were washed again with 1× PBS (pH 7.2) and resuspended in 400 µl 1× PBS. The mononuclear cells exhibiting increased fluorescence of oxidized DCF were measured by flow cytometry (FACSCalibur, Becton Dickinson, San Jose, CA) equipped with an argon ion laser (15 mW) tuned to 488 nm [23,24]. The fluorescence of DCF was collected in the FL1 channel, equipped with a 530/30 nm band pass filter. Fluorescence was measured in the long mode using CellQuest Pro software (BD Bioscience, San Jose, CA) and expressed as the geometrical mean fluorescence channel. Cells were gated on the basis of their characteristic morphology, i.e., forward scatter and side scatter of monocytes and lymphocytes. Acquisitions were performed on 10,000 gated events, while data analysis was performed with CellQuest Pro software (BD Bioscience).

### Measurement of total thiol

Total thiol or sulfhydryl groups (–SH) in PBMC and serum were measured spectrophotometrically by Elman’s method, modified by Hu et.al [25,26]. Mononuclear cells from each sample (5×10^6^) were homogenized in 100 µl cold buffer (100 mM Tris–HCL containing 1 mM EDTA, pH 7.5) for the estimation of PBMC total thiol level. Cells were centrifuged at 8.950 ×g for 15 min at 4 °C and the cell lysate soup from the supernatant was removed and diluted in Tris EDTA buffer to obtain the cell soup, which should contain 50 µg/µl of protein. Twenty microliters of diluted cell soup was used for intracellular thiol assay and expressed as µmol of thiol/mg of protein. Thiols present in the sample (serum/cell soup) reacted with 5, 5′- dithiobis-(2–nitrobenzoic acid) (DTNB) and formed a highly colored anion. According to the manual protocol, 25 µl of fresh serum was mixed with 1 ml Tris EDTA buffer (0.25 mmol/l Tris base, 20 mmol/l EDTA, pH 8.2) and the absorbance (A1) was measured spectrophotometrically (Halo DB-20; Dynamica, Salzburg-Mayrwies, Austria) at 412 nm, and in the next step, 12.5 µl of DTNB solution (10 mM in absolute methanol) was added into the solution. After 15 min incubation in ambient temperature, the absorbance (A2) was read again at 412 nm together with a DTNB blank. The concentration of total thiol in serum and PBMC samples were determined from the linear standard curve established by 0.2 to 1.6 mmol/l and 0.05 to 0.8 μmol/l of reduced glutathione as the sulfhydryl group standard.

### Statistical analysis

Data obtained from each sample group were expressed as (median [minimum to maximum range], mean±SD [standard deviation]). The means obtained from different sample groups were compared by the one-way analysis of variance test and the nonparametric Mann–Whitney U test. The parameters showing a statistically significant difference between the two groups were further analyzed using the two-tailed Student *t* test. To determine the correlation between two variables, Pearson’s product moment correlation coefficient was used. A value of p<0.05 was considered statistically significant. All statistical analyses were performed using GraphPad Prism software (version 5, 2007; Graph Pad software, San Diego, CA). Statistical analyses for sex distributions were evaluated by the *χ^2^* test by using STATA statistical software (version 8, Copyright 1984–2003, Stata Corporation, College Station, TX).

## Result

There was no statistical difference in age, sex distribution, duration of diabetes, body mass index, or blood pressure in PDR, NPDR, DNR, or HC individuals (Table 1). Fasting and postprandial blood glucose were elevated significantly among NPDR and PDR subjects compared to DNR and HC individuals (p=0.0001). HbA1c% was higher in NPDR patients (9.9±1.2%) compared to the DNR group (8.1±1.1%) and those with PDR (9.1±1.15%).

**Table 1:** Clinical characteristics of healthy control (HC), diabetic control (DNR), non-proliferative diabetic retinopathy (NPDR) and proliferative diabetic retinopathy subjects (PDR).

**Parameters**	**Data presentation mode**	**Variables**	**HC**	**DNR**	**NPDR**	**PDR**	**p value**
			(n=95)	(n=102)	(n=70)	(n=105)	
Sex		Male	51 (53.68%)	55 (53.92%)	39 (55.71%)	57 (54.28%)	0.9945
		Female	44 (46.32%)	47 (46.08%)	31 (44.29%)	48 (45.72%)	
Age (years)	Mean±SD		51.5±7.24	53.1±7.86	52±8.8	53.4±8.15	0.303
	Median (range)		52(35-65)	54(37-66)	49(38-66)	56(39-65)	
Duration of diabetes(years)	Mean±SD		———–	17.9±5.76	16.6±5.8	17.2±6.58	0.38
	Median (range)		———–	18 (8-27)	15 (8-29)	14 (10-32)	
BMI (kg/m^2^)	Mean±SD		24.2±3.98	25.3±4.78	25.9±4.64	25.6±4.37	0.061
	Median (range)		26 (18-29)	27 (18-32)	28 (19-31)	28 (18-31)	
Blood pressure (mmHg)	Mean±SD	Systolic	127.8±8.4	128.2±7.9	129.1±7.2	130±7.8	0.201
	Median (range)		129 (110-140)	130 (110-139)	130 (110-139)	131 (110-140)	
	Mean±SD	Diastolic	81.4±6.95	82.2±6.5	82.7±6.8	83.5±6	0.146
	Median (range)		85 (70-89)	86 (70-90)	86 (69-90)	86 (70-90)	
Blood glucose (mg/dl)	Mean±SD	Fasting	79±7.6	169±23.6	205.3±23.7	193.6±22.24	0.0001
	Median (range)		78 (65-98)	167 (129-235)	207 (145-268)	194 (146-256)	
	Mean±SD	Post Prandial	114.3±9.9	238±27.2	308.6±37.4	289.3±32.6	0.0001
	Median (range)		115 (80-134)	232 (190-289)	305 (247-370)	286 (208-359)	
HbA1c %	Mean±SD		4.7±0.7	8.1±1.1	9.9±1.2	9.1±1.15	0.0001
	Median (range)		4.8 (3.2-5.8)	7.9 (6.1-11.2)	10.1 (7.2-13)	9.2 (6.9-12.9)	

Table indicates the comparison of different groups enrolled in the present study shows no statistically significant difference for sex distribution, age, duration of disease, BMI and blood pressure. The HbA1c % and blood glucose value (fasting and post prandial) were increased in NPDR and PDR groups compared to HC and DC subjects and statistical analysis demonstrated a significant difference. Data were presented as Mean ± SD along with median (range). N indicates sample size. One way ANOVA test was done and p<0.05 was considered as minimum level of significance.

### Association of advanced glycation end products and N–epsilon carboxy methyl lysine with diabetic retinopathy occurrence

**Figure 1:**
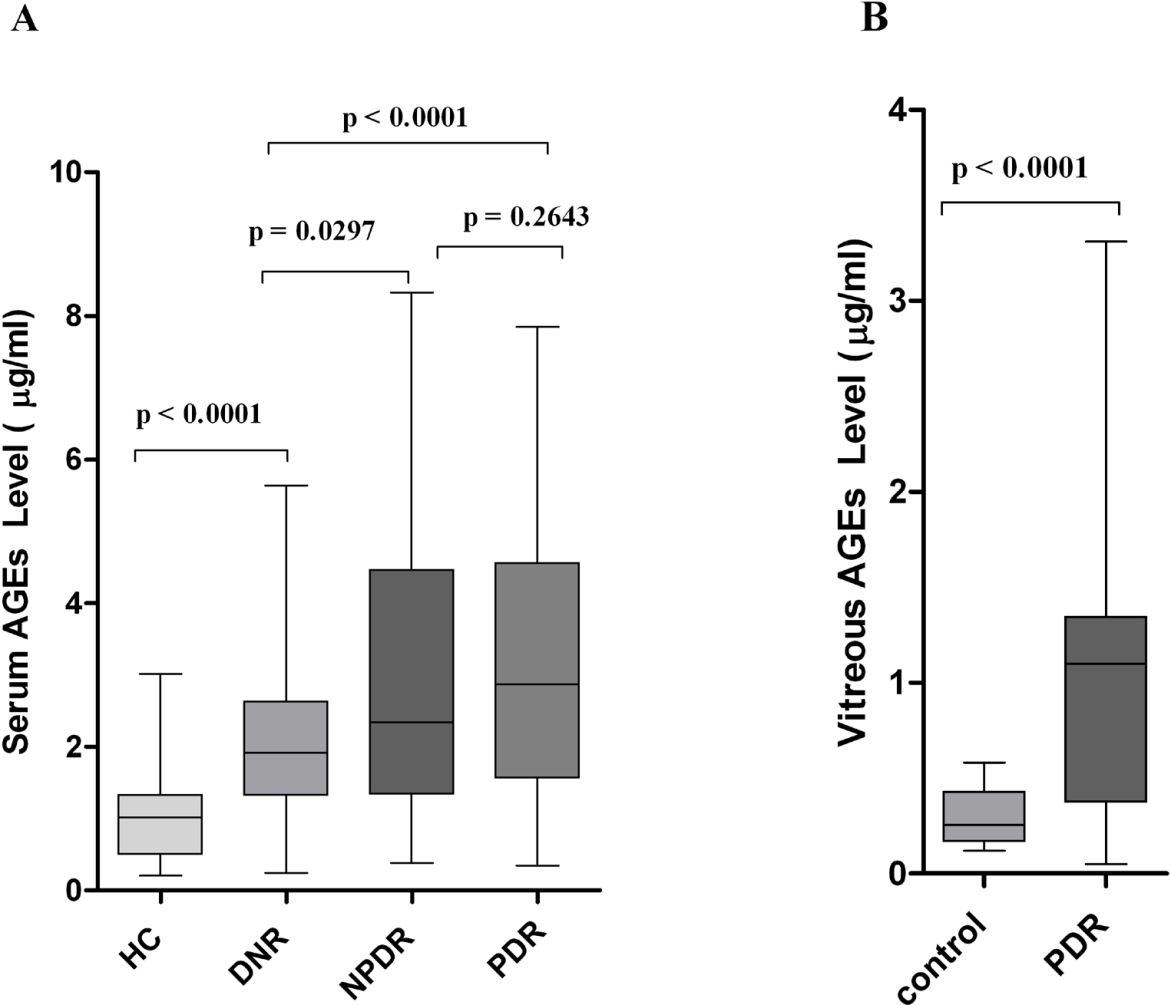
Serum and vitreous advanced glycation end-product levels among the different study groups. **A**: The box-and-whisker plot represents the median and minimum to maximum range of serum advanced glycation end-product (AGE) levels (µg/ml) among the different study groups. The serum AGE level was significantly elevated in subjects with proliferative diabetic retinopathy (PDR; p<0.0001) and nonproliferative diabetic retinopathy (NPDR; p=0.0297) compared to diabetes without retinopathy (DNR) subjects. Further, PDR subjects showed a higher level of serum AGEs than the NPDR group, but the difference was not statistically significant (p=0.2643). The level was found strikingly lower among healthy control (HC) subjects than those considered DNR (p<0.0001). Serum level of AGEs was measured in 105 subjects with PDR, 70 subjects with NPDR, 102 subjects with DNR and from 95 subjects considered as HC. **B**: The box-and-whisker plot represents the median and minimum to maximum range of vitreous AGE levels (µg/ml) among both the study groups. The vitreous level of AGEs was found to be significantly high among PDR subjects compared to the control group (p<0.0001). In this study, vitreous AGEs was measured among 45 subjects with PDR and 32 subjects considered as control.

The serum AGE level was significantly elevated in subjects with PDR (2.87 [0.35–7.85], 3.2±1.86 versus 1.92 [0.25–5.64], 2.16±1.29 µg/ml; p<0.0001) and NPDR (2.34 [0.39–8.32], 2.93±1.93 versus 1.92 [0.25–5.64], 2.16±.29 µg/ml; p=0.0297) compared to DNR subjects. Further, PDR subjects showed higher levels of serum AGEs than the NPDR group, but the difference was not statistically significant (p=0.2643). However, the level was found to be strikingly lower among HC subjects than those who were considered as DNR (1.02 [0.21–3.02], 1.05±0.61 versus 1.92 [0.25–5.64], 2.16±1.29 µg/ml; p<0.0001; Figure 1A).

The serum *N*^ε^-CML level was also elevated in a similar pattern to that of serum AGEs among PDR (0.69 [0.18–2.34], 0.89±0.53 versus 0.35 [0.11–1.31], 0.5±0.34 ng/ml; p<0.0001) and NPDR (0.91 [0.29–2.34], 1.05±0.51 versus 0.35 [0.11–1.31], 0.5±0.34 ng/ml; p<0.0001) subjects compared to the DNR group. However, NPDR subjects showed a significantly higher level of *N*^ε^-CML compared to the PDR group, and the difference was statistically significant (p=0.017; Figure 1B).

**Figure 2:**
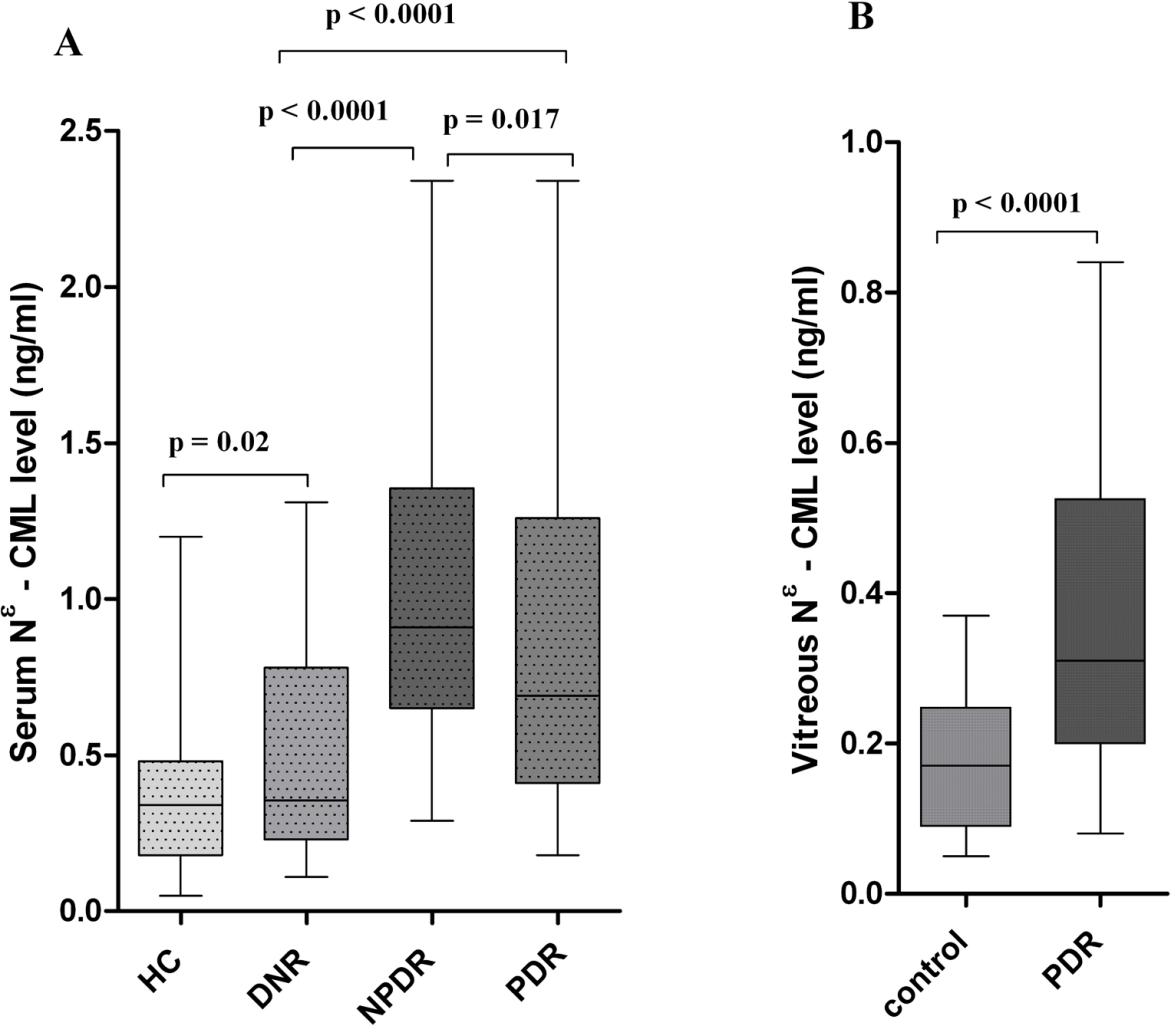
Serum and vitreous N-epsilon–carboxy methyl lysine levels among the different study groups. **A**: The box-and-whisker plot represents the median and minimum to maximum range of serum N-epsilon–carboxy methyl lysine (*N*^ε^-CML) levels (ng/ml) among the different study groups. The serum *N*^ε^-CML level was remarkably elevated among nonproliferative diabetic retinopathy (NPDR; p<0.0001) and proliferative diabetic retinopathy (PDR; p<0.0001) subjects compared to the diabetes without retinopathy (DNR) group. However, NPDR subjects showed significant higher levels of *N*^ε^-CML compared to the PDR group, and the difference was statistically significant (p=0.017). **B**: The box-and-whisker plot represents the median and minimum to maximum range of vitreous *N*^ε^-CML levels (ng/ml) among both study groups. The vitreous level of *N*^ε^-CML was found to be strikingly high among PDR subjects compared to the control group (p<0.0001).

The vitreous level of AGEs (1.1 [0.05–3.31], 1.04±0.75 versus 0.25 [0.12–0.58], 0.29±0.14 µg/ml; p<0.0001) and *N*^ε^-CML (0.31 [0.08–0.84], 0.35±0.19 versus 0.17 [0.05–0.37], 0.18±0.09 ng/ml; p<0.0001) were found to be significantly higher among PDR subjects than in the control group (Figure 1B and Figure 2B).

### Association of peripheral blood mononuclear cell reactive oxygen species level and diabetic retinopathy occurrence

**Figure 3:**
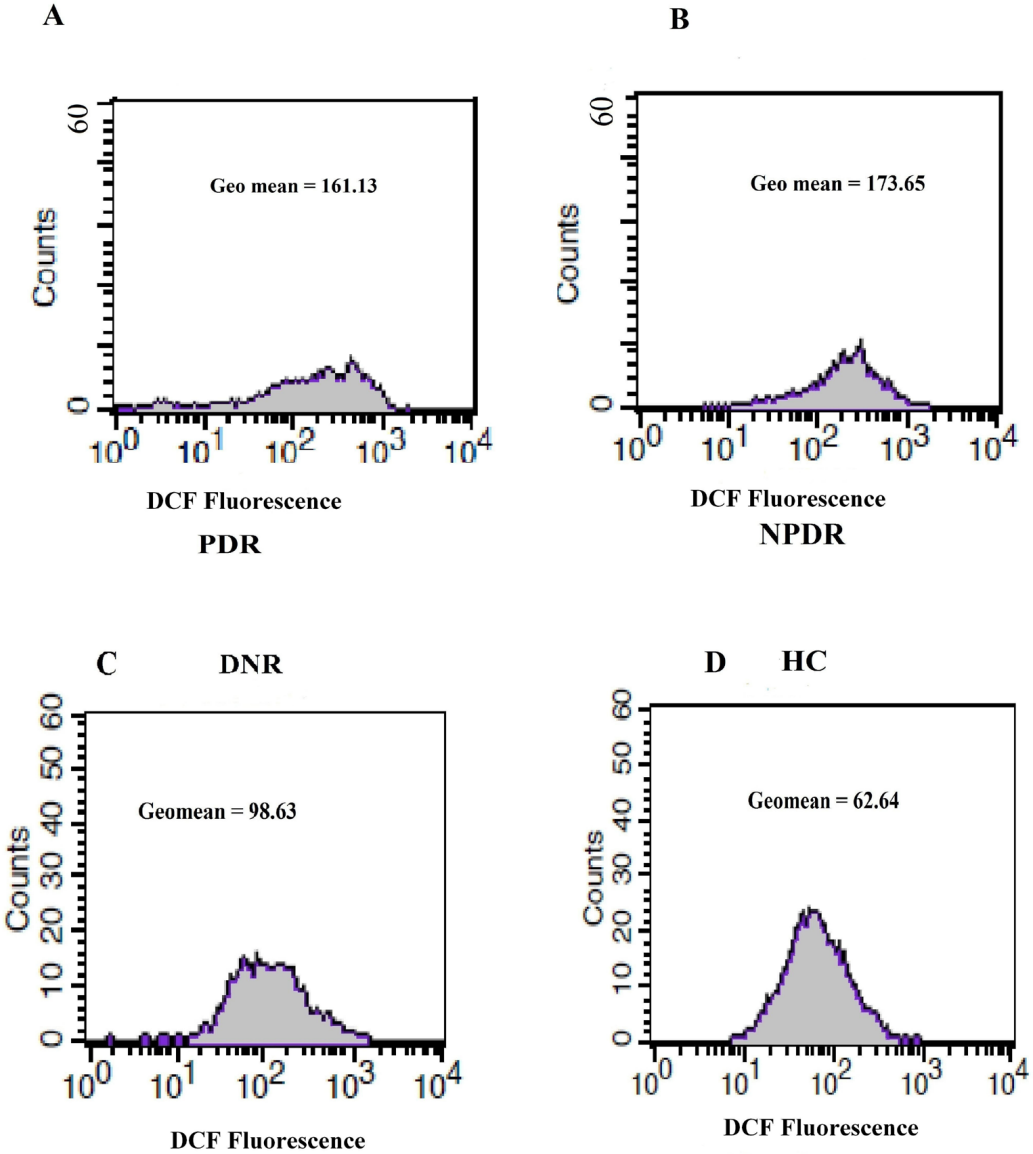
Histogram of peripheral blood mononuclear cell reactive oxygen species (PBMC ROS) level. The histograms in **A**, **B**, **C**, and **D** represent the geomean of PBMC dichlorofluorescein (DCF) fluorescence as a measure of ROS level among proliferative diabetic retinopathy (PDR), nonproliferative diabetic retinopathy (NPDR), diabetes without retinopathy (DNR), and healthy control (HC) subjects, respectively.

**Figure 4:**
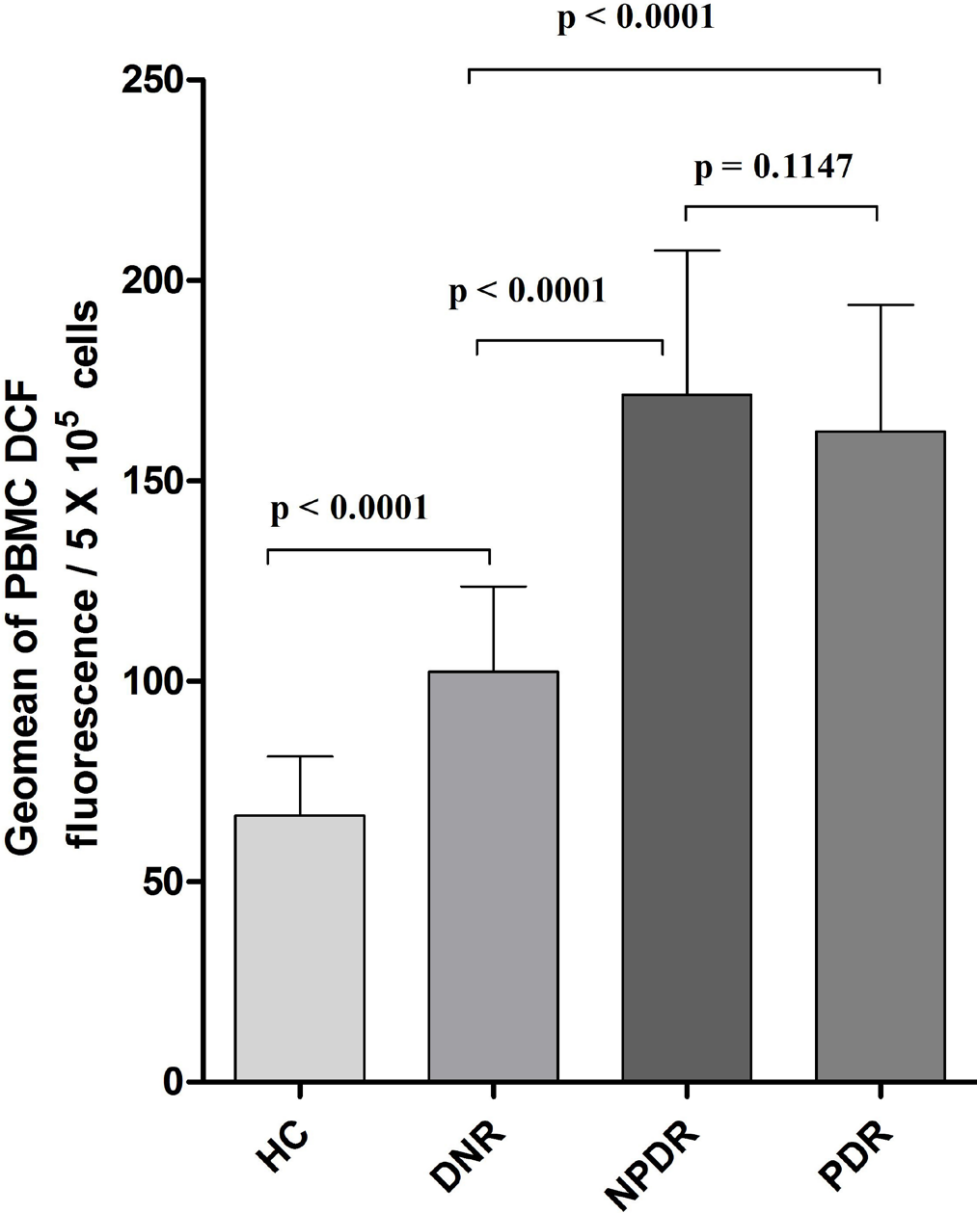
Peripheral blood mononuclear cell reactive oxygen species (PBMC ROS) levels in the different study groups. The bar columns represent the PBMC ROS level, which was expressed as mean±standard deviation (SD) of the geomean of DCF fluorescence/5x10^5^ cells among the study subjects. PBMC ROS production was found to be significantly high among nonproliferative diabetic retinopathy (NPDR) and proliferative diabetic retinopathy (PDR) subjects compared to diabetes without retinopathy (DNR) subjects (p<0.0001). A further increased trend of ROS production was observed among NPDR subjects than those with PDR. However, this production was not statistically significant (p=0.1147), whereas the level of ROS was found to be significantly lower among healthy control (HC) subjects even compared to the DNR group (p<0.0001).

The PBMC ROS level was expressed as the mean±SD of the geomean of DCF fluorescence/5x10^5^ cells among the study subjects (Figure 3A-D). PBMC ROS production was found to be significantly high among NPDR (170.4 [112.3–235.6], 171.39±35.98 versus 98.49 [62.34–147], 102.44±21.25; p<0.0001) and PDR (159.6 [91.42–234.5], 162.29±31.58 versus 98.49 [62.34–147], 102.44±21.25; p<0.0001) subjects compared to DNR subjects. Further, an increased trend of ROS production was observed among NPDR subjects in comparison to those were with PDR. However, this was not statistically significant (p=0.1147), whereas the level of ROS was found to be significantly lower among HC subjects even compared to the DNR group (65.24 [41.26–95.34], 66.48±14.7 versus 98.49 [62.34–147], 102.44±21.25; p<0.0001; Figure 4).

### Correlation between serum advanced glycation end products and peripheral blood mononuclear cell reactive oxygen species levels at different stages of diabetic retinopathy

**Figure 5:**
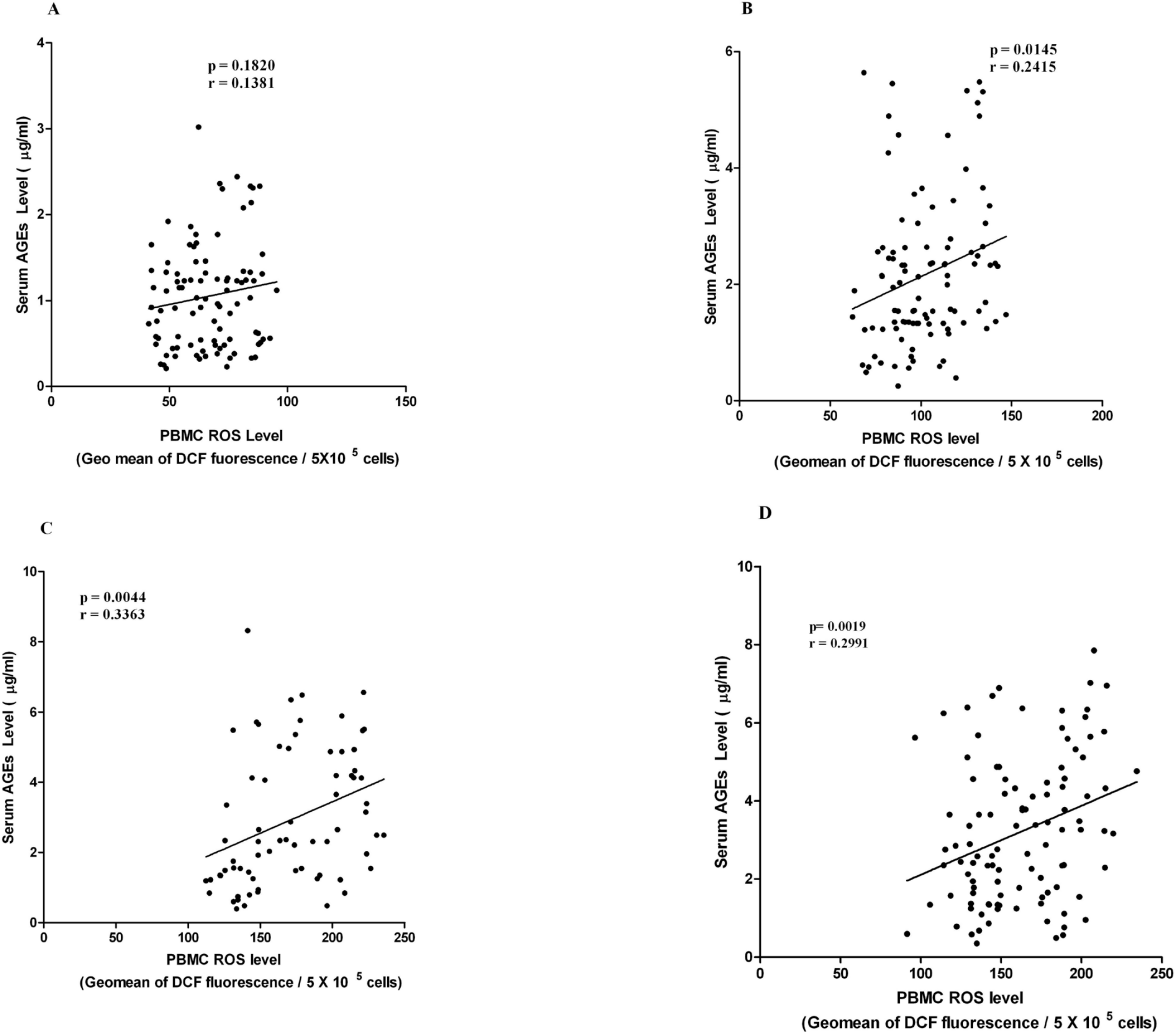
Correlation between serum advanced glycation end products (AGEs) and peripheral blood mononuclear cell reactive oxygen species (PBMC ROS) levels. **A**, **B**, **C**, **D**: The XY scatterplot represents the correlation between serum AGE level and PBMC ROS level among healthy control (HC), diabetic without retinopathy (DNR), nonproliferative diabetic retinopathy (NPDR), and proliferative diabetic retinopathy (PDR) subjects. A significant correlation was observed in between serum AGE levels and PBMC ROS levels among subjects with PDR (p=0.0019; r=0.2991), NPDR (p=0.0044; r=0.3363), and DNR (p=0.0145; r=0.2415), but not in HC individuals (p=0.182; r=0.1381).

A significant correlation was observed in between serum AGEs level and PBMC ROS level among subjects with PDR (p=0.0019; r=0.2991), NPDR (p=0.0044; r=0.3363), and DNR (p=0.0145; r=0.2415), but not in HC individuals (p=0.182; r=0.1381; Figure 5A-D).

### Correlation between serum *N*^ε^-CML and PBMC ROS levels at different stages of DR

**Figure 6:**
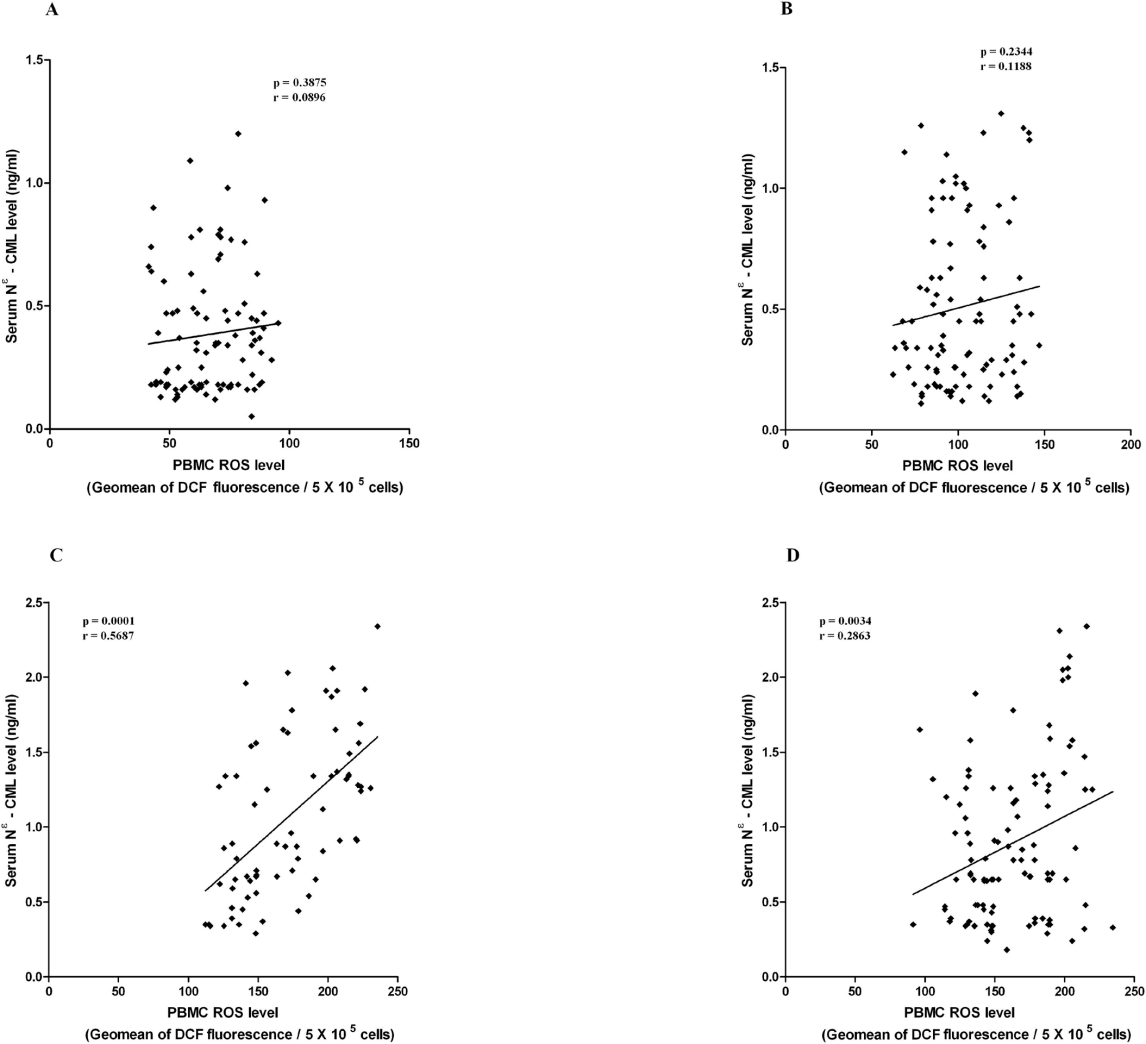
Correlation between serum N – epsilon carboxy methyl lysine (*N*^ε^-CML) and ” and peripheral blood mononuclear cell reactive oxygen species (PBMC ROS) level. **A**, **B**, **C**, **D**: The XY scatterplot represents the correlation between serum *N*^ε^-CML levels and PBMC ROS levels among healthy control (HC), diabetic without retinopathy (DNR), nonproliferative diabetic retinopathy (NPDR), and proliferative diabetic retinopathy (PDR) subjects. A significant correlation was observed in between serum *N*^ε^-CML levels and PBMC ROS levels among NPDR (p<0.0001; r=0.5687) and PDR (p=0.0034; r=0.2863) subjects, but no significant correlation was found in DNR (p=0.2344; r=0.1188) and HC individuals (p=0.3875; r=0.0896).

Significant correlation was observed in between serum *N*^ε^-CML level and PBMC ROS level among NPDR (p<0.0001; r=0.5687) and PDR (p=0.0034; r=0.2863) subjects. However, no significant correlation was found in DNR (p=0.2344; r=0.1188) and HC individuals (p=0.3875; r=0.0896; Figure 6A-D).

### Correlation of serum and vitreous levels of AGEs and *N*^ε^-CML at different stages of DR

A significant correlation was observed between serum AGE and *N*^ε^-CML levels among NPDR (p<0.0001; r=0.641) and PDR (p=0.0021; r=0.2974) subjects. However, no significant correlation was found in DNR (p=0.0771; r=0.1758) and HC individuals (p=0.1345; r=0.1547). In vitreous, the level of AGEs showed a significant correlation with the *N*^ε^-CML level among PDR subjects (p=0.0066; r=0.3992), but not in control subjects (p=0.3079; r=0.3992).

### Association of serum and PBMC total thiol level and DR occurrence

**Figure 7:**
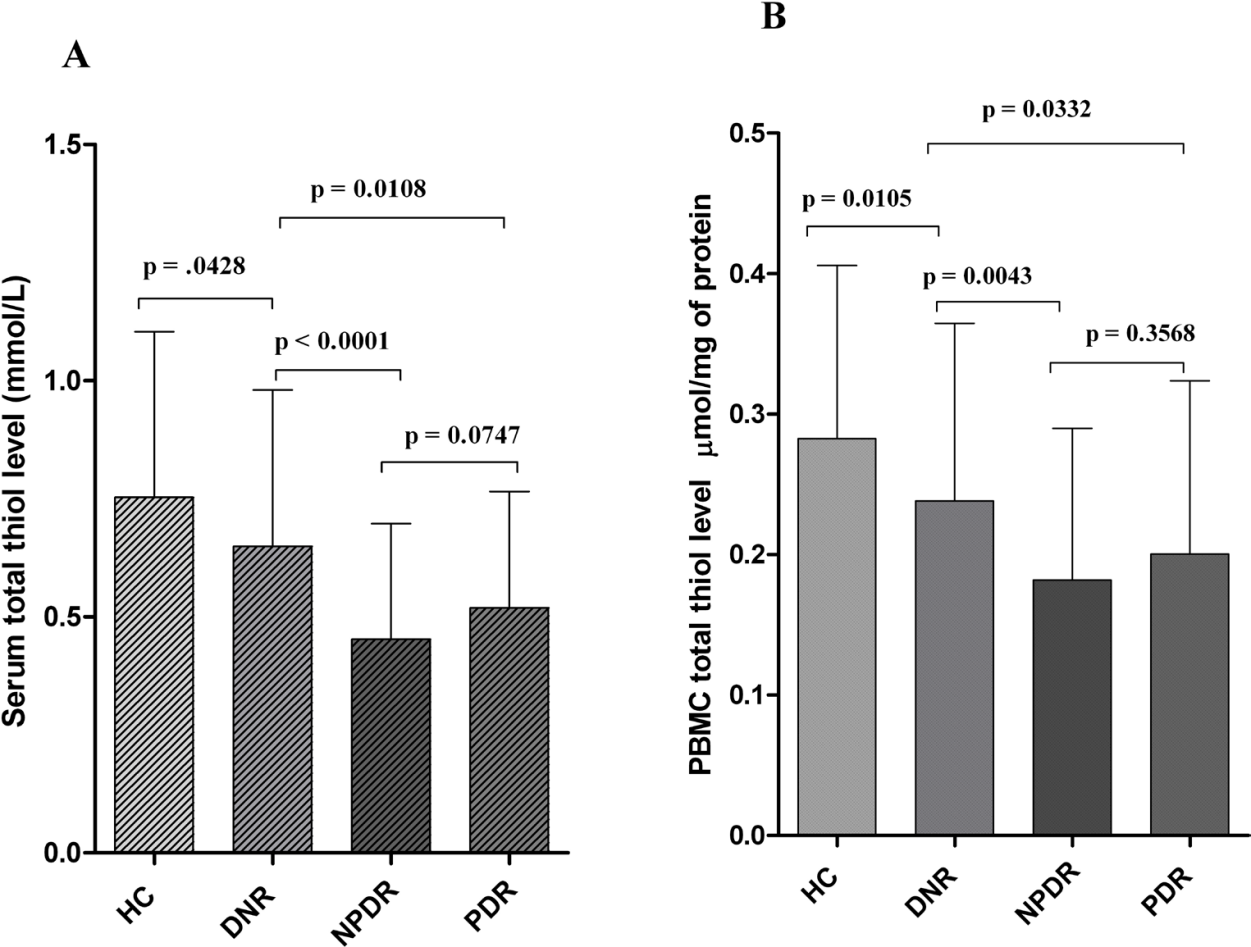
Serum and peripheral blood mononuclear cell (PBMC) total thiol level. **A**: The bar column represents the mean±standard deviation (SD) of total serum thiol level (mmol/l) among the different study groups. Total serum thiol levels were significantly decreased among nonproliferative diabetic retinopathy (NPDR; p<0.0001) and proliferative diabetic retinopathy (PDR; p=0.0108) subjects in comparison to those considered as having diabetes without retinopathy (DNR). The highest level of serum total thiol was found in healthy control (HC) subjects, even compared to the DNR group (p=0.0428). However, no significant difference was observed in total serum thiol level among NPDR and PDR subjects (p=0.0747). **B**: The bar columns represent the mean±standard deviation (SD) of PBMC total thiol levels (µmol/mg of protein) among the different study groups. The PBMC total thiol level was decreased significantly among NPDR (p=0.0043) and PDR (p=0.0332) subjects than those who were considered as DNR. The highest level of PBMC total thiol was found in HC subjects, even compared to the DNR group (p=0.0105). However, no significant difference was observed in PBMC total thiol levels between NPDR and PDR subjects (p=0.3568).

Serum and PBMC total thiol levels decreased significantly among NPDR (0.36 [0.15–1.05], 0.45±0.24 versus 0.57 [0.18–1.47], 0.64±0.33 mmol/l; p<0.0001 and 0.15 [0.03–0.47], 0.18±0.1 versus 0.24 [0.05–0.54], 0.23±0.11 µmol/mg of protein; p=0.0043, respectively) and PDR (0.52 [0.13–1.09], 0.51±0.24 versus 0.57 [0.18–1.47], 0.64±0.33 mmol/l; p=0.0108 and 0.17 [0.03–0.49], 0.2±0.12 versus 0.24 [0.05–0.54], 0.23±0.11 µmol/mg of protein; p=0.0332, respectively) subjects than those who were considered DNR. The highest level of serum and PBMC total thiol was found in HC subjects, even compared to the DNR group (0.68 [0.22–1.44], 0.75±0.35 versus 0.57 [0.18–1.47], 0.64±0.33 mmol/l; p=0.0428 and 0.27 [0.08–0.63], 0.28±0.12 versus 0.24 [0.05–0.54], 0.23±0.11 µmol/mg of protein; p=0.0105, respectively). However, no significant difference was observed in serum and PBMC total thiol level between NPDR and PDR subjects (p=0.0747 and p=0.3568, respectively; Figure 7A,B).

## Discussion

Microvascular pericyte dysfunction has been considered one of the earliest histopathological sign of DR. Apart from pericyte loss, some other key features like basement membrane thickening, blood barrier dysfunction, formation of microaneurysms, and capillary dropout are also observed in the retinal microvasculature in the early stages of DR [27,28]. These structural alterations herald irreversible retinal microvascular damage through EC proliferation associated with neovascularization [29]. Previous large-scale studies have emphasized the pathogenic role of AGEs in retinal pericyte dysfunction and DR-related complications [30-32]. To clarify this hypothesis, we have determined serum and vitreous total AGE and *N*^ε^-CML levels in subjects with NPDR, PDR, and DNR, as well as HC individuals.

Interaction of circulating AGEs with endothelial and pericyte RAGE transduces the signal for NADPH oxidases, which in turn produces oxidative stress. Generation of ROS through this signaling cascade perturbs cellular function by the upregulation of transcription factor nuclear factor kappa beta (NFκβ) [33,34]. AGEs may modify some vasoregulatory functions in retinal microcirculation and block the bioavailability as well as the antiproliferative activity of endothelium-derived nitric oxide [35,36]. AGEs promote DR and subsequent retinal ischemia by enhancing the process of platelet aggregation and fibrin stabilization, which are predisposed to the formation of microthrombuses in retinal microvessels [37,38]. Moreover, *N*^ε^-CML, the late oxidative product of AGEs, has been proposed as a biomarker of oxidation rather than glycation, and the level of N^ε^-CML reflects cellular metabolic disparity in subjects with type 2 DM [7-9].

Previous studies have suggested a detrimental role of AGEs in diabetic microvascular complications by demonstrating elevated serum total AGE levels among DR and nephropathy patients as compared to type 2 DM subjects without retinopathy and nephropathy and normal individuals [39,40]. Boehm et al. [9] reported that increased serum levels of CML were significantly associated with advance stages of retinopathy. In the present study, serum levels of total AGEs increased significantly in NPDR and PDR compared to DNR and HC subjects. However, the serum *N*^ε^-CML level was raised significantly in NPDR subjects even compared to PDR subjects, suggesting that the late oxidative product of AGEs, i.e., *N*^ε^-CML, would be expected to have pathogenic implications for retinal microvascular function in the early stages of DR. The significant increase of total AGEs in PDR subjects indicates that the total AGEs are the major contributor in EC and pericyte dysfunction in the proliferative stages of DR, and might be the key mediators for the development of proliferative retinopathy from the nonproliferative stage.

Total AGE and *N*^ε^-CML concentrations were found to be significantly higher in PDR vitreous compared to vitreous from normal individuals without type 2 DM. This represents the first time that *N*^ε^-CML was measured from the vitreous body of PDR subjects. Vitreous is a hydrated gel matrix composed of a complex network of cross-linked collagen fibers [41]. Stitt et al. [42] reported that cross-linking of AGEs with the vitreous collagen network leads to liquefaction and several abnormalities of vitreous in subjects with type 2 DM. Our study supports the theory that AGEs are toxic to retinal pericytes and activate a subsequent signaling cascade for cellular apoptosis that might have an aggravative impact on blood-retinal barrier integrity [31]. An accumulation of AGEs along with *N*^ε^-CML in PDR vitreous might be due to blood-retinal barrier dysfunction, which is caused by extensive loss of retinal pericytes and EC.

We found a significant correlation between *N*^ε^-CML and AGEs in serum and vitreous samples of PDR subjects. However, the relatively highly significant correlation between *N*^ε^ -CML and AGEs in serum samples of NPDR subjects caused us to speculate that *N*^ε^ -CML, the key molecule, which is significantly associated with NPDR occurrence and rises remarkably with AGE levels in preproliferative and proliferative stages of retinopathy. Yet again, this assessment has clearly revealed that elevated levels of *N*^ε^-CML along with AGEs commence the process of retinopathy, and their prolonged elevation worsens the process of retinopathy in type 2 DM.

Persistent hyperglycemia induces oxidative stress when ROS are overproduced or endogenous antioxidant systems are impaired [43,44]. Members of the ROS family have divergent effects on EC function, such as cell proliferation, migration, angiogenesis, and modulation of extracellular matrix production and breakdown [45]. Increased production of ROS is also due to the uncoupling of NADPH oxidases and upregulates the transcription factor NFκβ, which in turn transcribes its target genes and leads to the expression of vascular cell adhesion molecule 1, intracellular adhesion molecule 1, E selectin, vascular endothelial growth factor, and likely proinflammatory cytokines, including interleukin-6, interleukin-α, and tumor necrosis factor α [11,46]. A growing body of evidence suggests that these growth factors, along with cytokines, play an important role in the development and progression of DR in type 2 DM [15,47]. Nam et al. [48] reported that increased PBMC ROS generation is strongly involved in the pathogenesis of diabetic nephropathy via the activation of NFκβ. Isoni et al. [49] found that ROS production by PBMCs in type 2 DM subjects was approximately 1.6 times higher than in healthy individuals. In vitro studies on bovine retinal pericytes and aortic EC have demonstrated that ROS generation from NADPH oxidase plays a key role in the intracellular metabolic disparity and apoptosis of retinal capillary pericytes [50]. In our study, we observed that the PBMC ROS level increased significantly in NPDR and PDR subjects compared to DNR and HC subject. In addition, an increased trend of ROS was observed among NPDR subjects compared to PDR subjects, but the difference was not statistically significant. However, our observations suggest that increased PBMC ROS production may cause early pathological changes in the retinal capillaries of NPDR subjects, further rendering retinal capillaries vulnerable to further sustained oxidative stress and resulting in the development of PDR.

We observed that PBMC ROS levels correlated significantly with serum *N*^ε^-CML and AGEs in NPDR and PDR subjects. An even more significant correlation was observed between serum AGE and PBMC ROS levels among DNR subjects. However, the strongest correlation was seen between serum *N*^ε^-CML and PBMC ROS levels in NPDR subjects; this was extremely statistically significant, elucidating that *N*^ε^-CML might be the key molecule to trigger the production of ROS and thereby activate intracellular downstream signaling molecules, which are intimately associated with development of retinopathy in type 2 DM.

Based on the above discussion, it may be hypothesized that the elevated levels of serum and vitreous AGEs among PDR subjects compared to NPDR group suggests that the detrimental effect of AGEs is intimately associated with the severity of retinopathy. Mainly, the interaction of circulating AGEs and RAGE has attracted the support for the notion that EC homeostasis is perturbed among DR subjects with elevated levels of AGEs [51]. Large-scale studies have reported that intracellular AGE–RAGE interaction–mediated ROS generation induces monocyte chemoattractant protein 1, plasminogen activator inhibitor 1, and increased vascular endothelial growth factor messenger RNA expression by NFκβ activation via the Ras mitogen-activated protein kinase pathway in retinal microvascular ECs. ROS mediated increased upregulation of these downstream molecules further aggravates and again promotes the process of retinopathy by favoring retinal angiogenesis, thrombogenesis, and inflammation [37,52,53]. Following the results of the above reports, we believe that inhibition of AGE formation or blockage of AGE–RAGE interaction–mediated downstream signaling pathways might be a potential therapeutic approach for preventing the proinflammatory roles of AGEs among NPDR and PDR subjects who already have an elevated level of circulating AGEs.

The total thiol pool constitutes the majority of the total body antioxidants and plays a major role in defense against ROS [19,54]. Baskol et al. [20] observed that serum thiol levels were significantly lower in DR subjects compared to those without DR and controls. Our study showed a significant decrease in serum and PBMC thiol levels in NPDR and PDR subjects compared to DNR and HC subjects. The decreased level of total thiols in the PBMCs and serum of NPDR subjects could be due to increased oxidation of –SH groups due to oxidative stress, which was reflected by the increased ROS level in NPDR subjects. This deprived antioxidant state in NPDR and PDR patients may explain the increased susceptibility of retinal microvasculature to oxidative injury in poorly controlled patients with type 2 DM.

In conclusion, it may be said that elevated levels of serum and vitreous *N*^ε^-CML and AGEs are associated with an increased occurrence of DR in subjects with type 2 DM. In particular, *N*^ε^-CML is linked with the early development of retinopathy, which might accelerate intracellular ROS production, and total AGEs may influence the proliferative changes in retinal capillaries of NPDR subjects. As a whole, sustained oxidative stress induced by increased ROS production under an antioxidant-deprived state among NPDR subjects with poor glycemic control might be the key regulator in the development of PDR.
